# Finding harmony within dissonance: Engaging patients, family/caregivers and service providers in research to fundamentally restructure relationships through integrative dynamics

**DOI:** 10.1111/hex.13063

**Published:** 2020-06-11

**Authors:** Gillian Mulvale, Jenn Green, Ashleigh Miatello, Ann E. Cassidy, Terry Martens

**Affiliations:** ^1^ DeGroote School of Business McMaster University Hamilton ON Canada; ^2^ Health Policy PhD Program McMaster University Hamilton ON Canada; ^3^ Hamilton ON Canada

**Keywords:** experience‐based co‐design, health system improvement, integrative dynamics, mental health research, transition‐age youth, youth and family engagement

## Abstract

**Background:**

Deeply divided ideological positions challenge collaboration when engaging youth with mental disorders, caregivers and providers in mental health research. The integrative dynamics (ID) approach can restructure relationships and overcome ‘us vs them’ thinking.

**Objective:**

To assess the extent to which an experience‐based co‐design (EBCD) approach to patient and family engagement in mental health research aligned with ID processes.

**Methods:**

A retrospective case study of EBCD data in which transitional‐aged youth (n = 12), caregivers (n = 8) and providers (n = 10) co‐designed prototypes to improve transitions from child to adult services. Transcripts from focus groups and a co‐design event, co‐designed prototypes, the resulting model, evaluation interviews and author reflections were coded deductively based on core ID concepts, while allowing for emergent themes. Analysis was based on pattern matching. Triangulation across data sources, research team, and youth and caregiver reflections enhanced rigour.

**Findings:**

The EBCD focus group discussions of touchpoints in experiences aligned with ID processes of acknowledging the past, by revealing the perceived identity mythos of each group, and allowing expression of and working through emotional pain. These ID processes were briefly revisited in the co‐design event, where the focus was on the remaining ID processes: building cross‐cutting connections and reconfiguring relationships. The staged EBCD approach may facilitate ID, by working within one's own perspective prior to all perspectives working together in co‐design.

**Conclusion:**

Researchers can augment patient engagement approaches by applying ID principles with staged integration of groups to improve relations in mental health systems, and EBCD shows promise to operationalize this.

## 1 BACKGROUND

Experience‐based co‐design (EBCD)^1^, ^2^, ^3^ is a best practice approach to mental health system improvement^4^ that grounds service design in the experiences of service users, their family or other caregivers (caregivers), and service providers (providers), who work together to co‐design service improvements.^1^, ^2^ EBCD is increasingly being applied in mental health research^5^, ^6^, ^7^, ^8^ as it strongly aligns with a recovery orientation^9^, ^10^, ^11^, ^12^, ^13^ by placing lived experience at the centre of mental health service improvement.^14^

Deeply entrenched ideological divides among service users, caregivers and providers may pose a challenge to collaborative patient and family engagement in mental health research. Key tensions^14^ include the emphasis on treatment vs promoting positive mental health,^15^, ^16^, ^17^, ^18^, ^19^, ^20^ extent of family involvement^21^, ^22^ and adopting a recovery vs biomedical approach.^23^ Issues are exacerbated for transitional‐aged (16‐25 years) youth and their families, who often experience abrupt service termination at age 16 or 18, long waits before transitioning to adult services and culture shock upon entry into adult services.^21^, ^24^, ^25^, ^26^, ^27^ Often youth feel disempowered due to stigma and age, caregivers feel shut out of adult services, and providers feel defensive due to system constraints. These collective experiences may result in deep mistrust of services^28^ and emotionally charged interactions between the perspectives.^2^, ^5^, ^28^ Engagement processes may break down if the historical relations between perspectives are not considered.

In the diagnostic phase of EBCD,^1^, ^29^ researchers engage service users, caregivers and providers to understand their service experiences,^1^, ^2^, ^29^ often through ethnographic observation and individual interviews to understand the emotional highs or lows where experiences are powerfully shaped.^1^, ^29^, ^30^ These ‘touchpoints’ are discussed in separate focus groups to determine improvement priorities for each perspective, often by experience mapping. In the second, intervention phase, mixed participant groups collaboratively generate visual prototypes^31^, ^32^ of service improvements through a facilitated ideation process^7^, ^29^, ^30^ at a co‐design event. Often a trigger video (compiling participants' perspectives) kicks off co‐design discussions.^7^, ^30^ Implementation and summative evaluation phases follow, alongside formative evaluation. While helpful resources outline EBCD procedures,^5^, ^33^ how to embrace discord while fostering harmonious co‐design is less clear.

Shapiro^34^ states that traditional approaches to negotiation typically fall short in situations of emotionally charged conflict where issues of identity are at stake, by failing to change fundamental group dynamics. He argues that fostering integrative dynamics (ID)—the ‘emotional forces that pull you toward greater connection’—can help to overcome conflict and heal broken relations (p.134).^34^ Shapiro argues that people can move beyond opposing perspectives and the ‘duality of us vs them’ (p.134),^34^ by focusing on the shared issue or problem to be worked through,^35^ even when the core identities of different groups ‘may *feel* completely incompatible’ (p.131).^34^ Adversarial relations become collegial through an emotionally intense process (relational conversion) that shifts the emotional space towards a cooperative, compassionate and open communal mindset, allowing each group to imagine new creative approaches in how to relate to one another^34^, ^35^ with the most stable connection being ‘transcendent unity’ as a state of mind (p.134).^34^

Throughout our research programme applying EBCD in youth mental health, our research team has witnessed such mindset shifts. In these moments, the co‐design atmosphere is dramatically altered from perceived separation, power imbalance, wariness and mistrust, to mutual understanding and respect, where each perspective values the other's contributions in co‐designing effective improvements. Deliberations at an international symposium^28^ echoed these observations; however, exactly what enabled such shifts is not well understood and is beginning to be explored.^36^

Our proposition is that the relational conversion discussed in ID can be achieved via EBCD processes during mental health engagement activities. We conducted a retrospective case study in order to test this proposition.

## 2 METHODS

### 2.1 Retrospective case study

The *my*Protocol EBCD study, conducted from February to May 2019, was selected as an illustrative case^37^ for retrospective analysis wherein the risk of emotional conflict was high, yet relational conversion appeared to be achieved, even though ID concepts were not a part of the study design. The *my*Protocol objective was to inform the development of a transitions protocol from child to adult mental health services for youth aged 16‐25, involving a Working Group (WG) of 26 service organizations in a Local Health Integration Network (LHIN) in Ontario, Canada. The retrospective analysis, conducted between June and December 2019, explored whether and if so, how, ID concepts contributed to relational conversion in the *my*Protocol EBCD processes.

### 2.2 Conceptual framework

**FIGURE 1:**
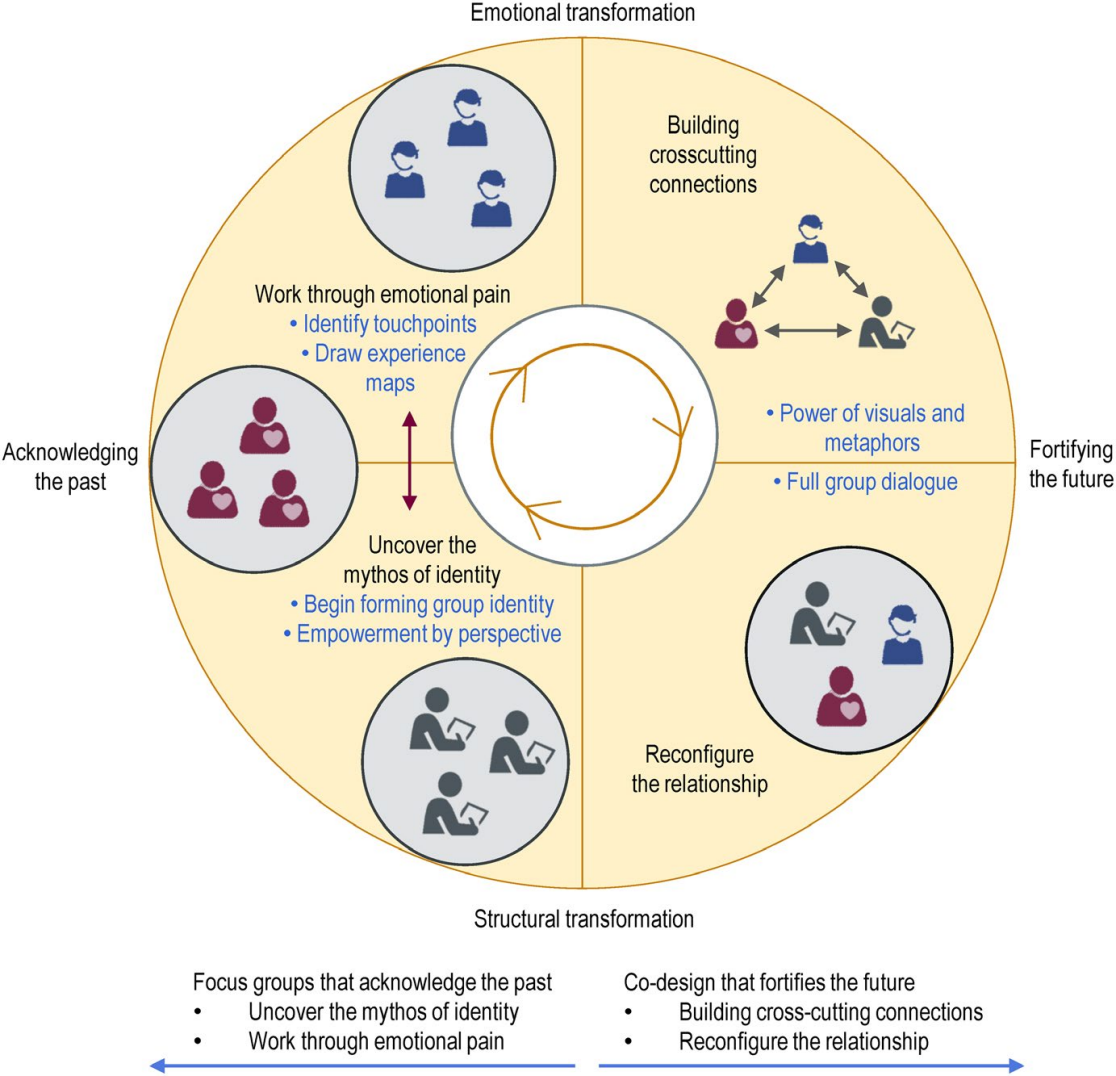
ID processes within EBCD focus groups and co‐design event

Consistent with recommended practice in case study research,^37^ we adopted a guiding conceptual framework (ID model)^34^ and used pattern matching of data to the core concepts of this framework.^37^ Figure 1 depicts the four key iterative processes within an ID approach to resolving identity‐based differences. The first involves uncovering how each side views themselves in relation to others, referred to as their ‘mythos of identity’. The second is to acknowledge the narratives of each group, working through emotional pain. The third builds authentic connections among participants. The fourth recasts the relationship among groups as ‘a mutually affirming narrative’ resulting in more harmonious interactions that ‘strive toward transcendent unity’.^34^

### 2.3 EBCD participants and recruitment

There were a total of 30 participants who attended either a focus group (n = 24) or the co‐design event (n = 25) or both in the *my*Protocol process (See Table 1). Participants were recruited by the research team with assistance from WG members. Balanced numbers of participants across perspectives and LHIN subregions were invited to participate.

**TABLE 1:** Overview of participants who attended *my*Protocol process

Participant type	Focus group	Co‐design event	Either
Youth	9	11	12
Caregiver	7	7	8
Service provider	8	7	10
Total	24	25	30

### 2.4 Data sources

Table 2 provides an overview of the data sources. Three 2‐hour web‐based focus groups were held by perspective, followed by a 5‐hour in‐person co‐design event. Key themes were synthesized into the ‘continuity vortex model’ which is a guiding framework for the transitions protocol. All participants were invited to provide feedback on the draft model by email or through an online focus group, and revisions were incorporated. In addition, 23 evaluation surveys (79.3% completion rate) and 9 evaluation interviews (3Y/3CG/3P) were completed about participants' EBCD experiences. Focus group and co‐design event discussion transcripts illustrate how ID model elements were encompassed in the EBCD stages. Evaluation interviews demonstrate alignment of the overall experience with ID concepts. A semi‐structured guide was used in the author's reflective discussion to probe directly about ID processes and principles (see Appendix 1). Written responses to the question ‘*What would you tell someone about the co‐design process you experienced that resulted in the Continuity Vortex model?’* were provided by one youth (AC) and one caregiver (TM) co‐author.

**TABLE 2:** Overview of data sources

Data sources	Participants	Original objective/outputs
1. 3 focus groups (FG)—youth, family/caregiver and service providers	24 (9 youth, 7 caregivers and 8 providers)^a^	To identify and validate touchpoints in experiences/experience summaries by perspective/transcripts from discussions
2. Co‐design event (CD)	25 (11 youth, 7 caregivers and 7 providers)^a^	To co‐design service elements of transitions protocol/transcripts from discussions and 3 prototypes to improve service transitions: guiding principles improved youth friendliness protocol elements
3. Continuity vortex model (CV)	Developed by research team with written feedback (n = 8; 1 youth, 3 caregivers and 4 providers) and online focus group (n = 3; 1 youth and 2 caregivers)	An overarching model that combines key themes from co‐design event and focus groups as a basis for future work to refine and implement a transitions protocol for the LHIN
4. Evaluation interviews (EI)—*my*Protocol study	9 (3 youth, 3 caregivers and 3 providers)	To understand and improve experiences of engagement processes (focus groups and co‐design event)
5. Author reflection discussion (AR)	1 youth, 1 caregiver and 2 research team members	To explore extent to which ID principles and processes were experienced by EBCD participants
6. Written reflections (WR)	1 youth and 1 caregiver	To reflect on overall experience from respective perspectives.

^a^ Same participants across the focus groups and co‐design events with minor variations due to participant availability at either event.

### 2.5 Data management and analysis

Audio recordings were transcribed verbatim by a professional transcription service and de‐identified, for example Y09FG [(Youth/Y; Caregiver/CG; Provider/P)/number/ source]. Data analysis was iterative. The research team (GM, JG and AM) deductively coded EI data using the categories of the ID model, searching for confirming and disconfirming evidence, allowing for emergent themes and discussing discrepancies until consensus was reached. Two authors (GM and AM) coded the AR, WR and CV data using the same codebook. Following reviewer suggestions, we revised the codebook (Appendix 2) to fully explore ID processes across EBCD stages and applied it to an expanded data set (FG and CD), triangulating across data sources to enhance rigour.^37^, ^38^, ^39^ The study received ethics approval from the Hamilton Integrated Research Ethics Board (study #1982). We used the COREQ checklist as a tool to review and guide the reporting of our methods and findings of this manuscript.

## 3 FINDINGS

### 3.1 Core processes of integrative dynamics achieved through EBCD processes

**FIGURE 2:**
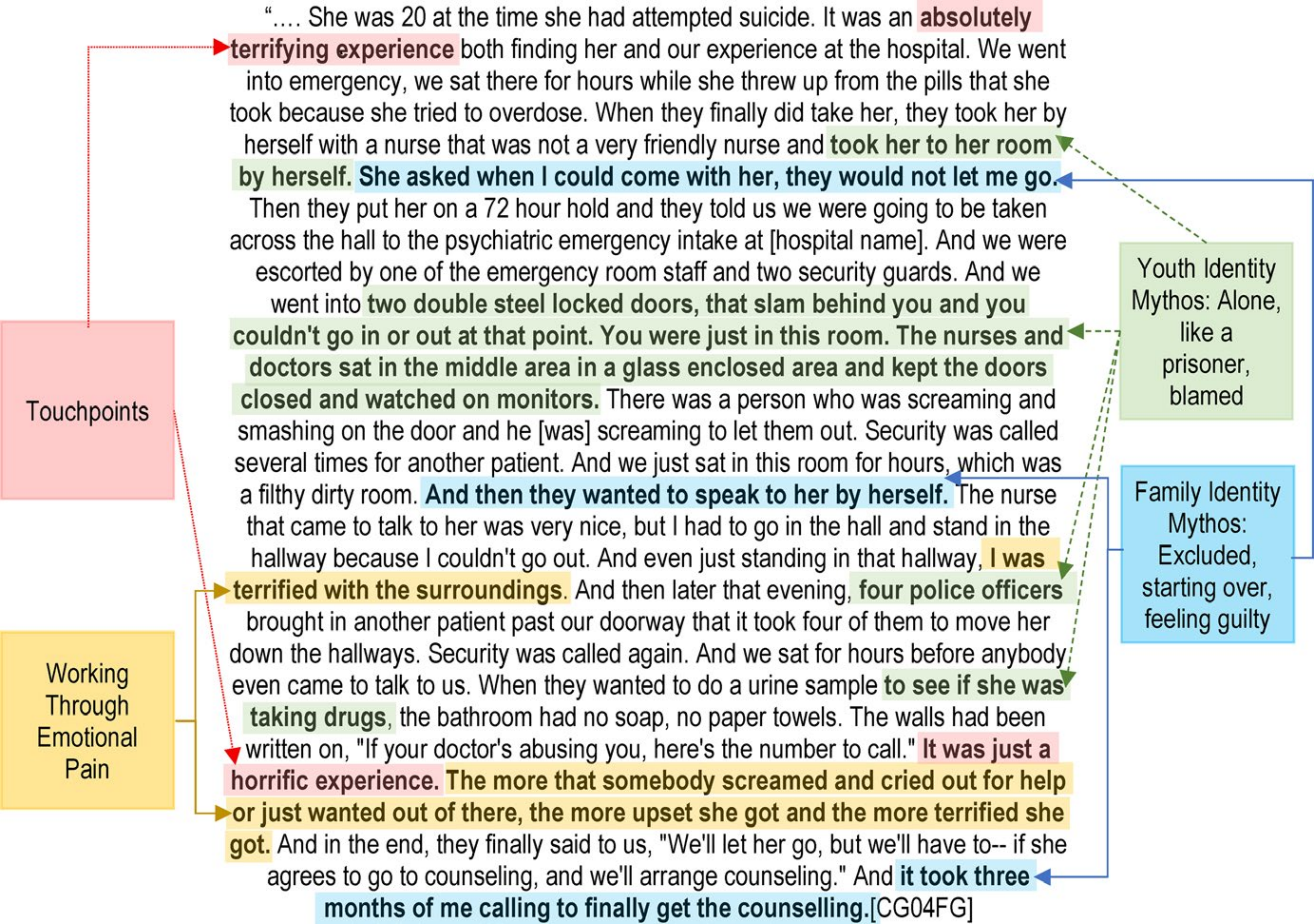
Identifying mythos of identity and working through emotional pain when discussing a touchpoint

Two ID processes (uncovering the mythos of identity and working through emotional pain) were primarily associated with the separate focus groups in the first stage of our EBCD process. The remaining ID processes (building cross‐cutting connections and reconfiguring the relationship) were primarily associated with the co‐design stage (see Figure 2). We present our findings for each stage in turn.

#### 3.1.1 Acknowledging the past at online focus groups

**FIGURE 3:**
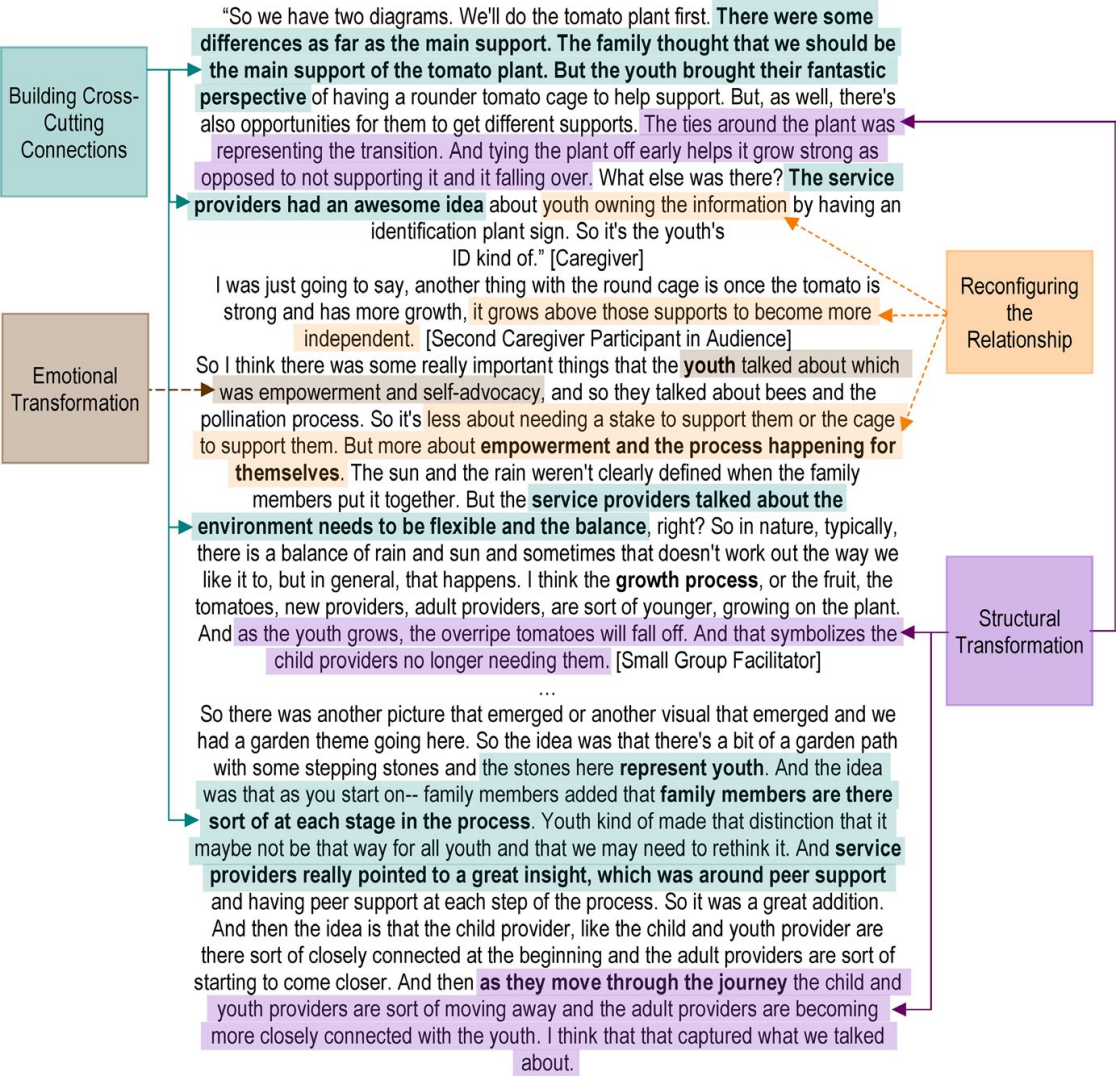
Co‐design to achieve emotional and structural transformation by building cross‐cutting connections and reconfiguring the relationship

The core ID processes of uncovering the mythos of identity and working through emotional pain were evident during the sharing of touchpoints during the focus groups. Figure 3 illustrates the identification of a touchpoint—a hospital visit for a youth suicide attempt—shared at the caregiver focus group, as the participant shares emotional pain and uncovers core elements of the caregiver and youth identity mythos.

##### Uncover the mythos of identity

For **youth**, feeling frightened, alone and blamed by the system were repeatedly heard. Some felt like prisoners, who were not even allowed visitors,

I was put in [hospital] psych ward … when I was 15. …So those three and a half weeks, I was not allowed to see my parents. My workers didn't come and visit me. No, I was literally locked in the psych ward. [Y06FG]

Others described feeling blamed for being manipulative when admitting suicidal ideation,

… That was essentially me saying please don't leave me alone. And they looked at me and said, “I'm sorry, we don't tolerate threats here”. And they closed the door and walked away. [Y03FG]

Youth also felt blamed for not trying hard enough to find services during the transition period, and were asked ‘Well why didn't you come and get help when you were 15?… were you not serious about getting help?’, when in fact there were ‘simply no support systems in place for anyone to get to at that point.’ [Y06FG]

**Caregivers** described being excluded from their youth's care, particularly as the youth aged,

I felt like I was out of the loop from the time he was 14 with the doctors because of the privacy act with my son's rights. It was like, “How can a 14‐year‐old have so many rights?” and as a parent I didn't feel that I had any. [CG07FG]

Like the mythical Sisyphus, families felt condemned to keep starting over and over again in trying to find the right care for the youth,

… they can't help them if the care isn't consistent. And so, we wait and we find care. [Then] it's taken away. And so we have to start all over again. So maybe he's made a little progress, but then we have to start looking again for something else to help.… – start all over again – start all over again … [CG05FG]

A related theme was the pressure on families, ‘… the toll it takes on you and your family. It's almost never‐ending. It's exhausting’. [CG05FG] This is compounded by guilt felt when other family members say, ‘it's all about her,’ [CG04FG] and when services are not helpful,

Because we've said to our child, “We'll take you here; they'll help you,” and they don't. They just, in essence, make it worse. And then we, on top of everything else, have all this guilt because our child thinks we lied to them… [CG05FG]

**Providers** described trying to assist youth through transitions, but having their hands tied by a disconnected system,

…they kind of hit 18, they age out, and service is done. I know we've done our best to kind of reach out and try to foster that relationship [across services], but it isn't happening at the rate we expected. [P05FG]

Very different care models between child and adult services also hampered transitions,

…because the child system and adult system are set up so differently … So then, youths are kind of cut off in the middle of treatment sometimes, and there's not something to continue with on the other side ‐‐ it's a different model completely sometimes. [P03FG]

##### Working through emotional pain

For **youth,** the focus group offered a place to share feelings of overwhelm, frustration and anger, with others who had similar transitions experiences. One youth described such feelings when attending an adult group they were not ready for,

I wasn't ready, myself, to have CBT. I would go into every session, and I would cry and cry because I was so scared, like I didn't do the homework…I felt like I was letting everyone down…my counselor would ask me, “Are you sure you want help?” And I was so angry. I was like, “Of course, I want help. That's why I'm here." But it's so hard to do everything when I'm feeling like this too. [Y02FG]

The focus group was also cathartic for **caregivers**. One caregiver was moved to tears sharing feeling insulted when a service provider seemed to suggest their daughter was a ‘lost cause’,

We took a giant step back in child services because we had a psychiatrist that told our daughter that there was nothing they could do for her… we were very, very upset. So it wasn't only getting services … We were going backwards and our daughter took that to heart and still holds it to heart. She has a hard time trusting anybody because of that statement. [FM03FG]

Hearing similar experiences from other caregivers was reassuring,

… the support that I felt from other families or caregivers. Listening to what they had been through, the roadblocks they had experienced reassured me that I was not alone. Their support and comfort helped me through difficult moments. [CG‐AR]

For **providers**, there was less emotional pain to work through; however, there was considerable frustration with the lack of responsiveness of the system.

…[the] waitlist is so long that they're not able to get into services at all, so the referral source will say, “You know what? It's not even worth completing the referral.” And then we have nowhere to send them. [P07FG]

#### 3.1.2 Fortifying the future at the co‐design event

**FIGURE 4:**
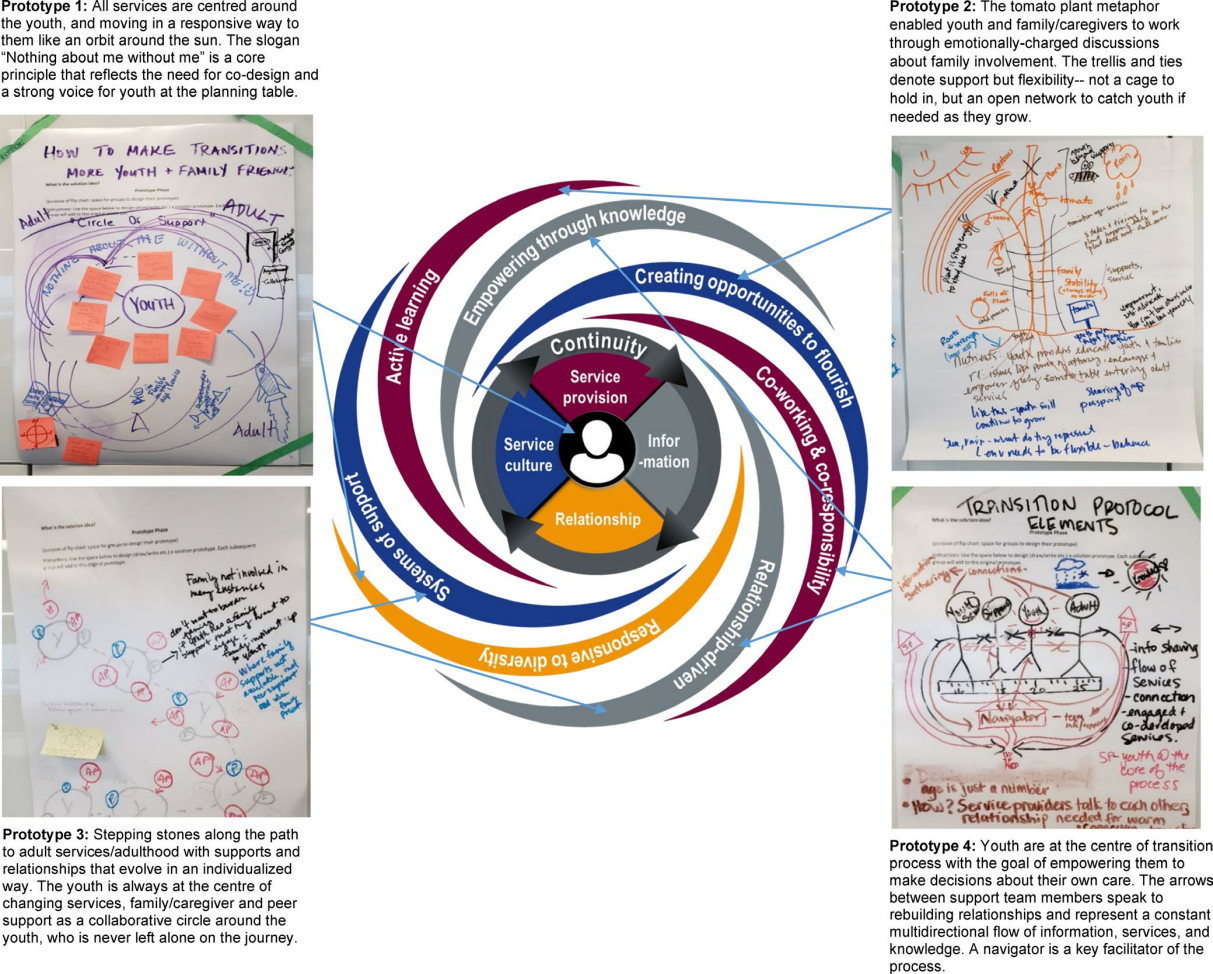
The continuity vortex model derived from prototypes of restructured relationships

At the co‐design event, emphasis shifted from acknowledging the past to fortifying the future. Figure 4 illustrates how themes of building cross‐cutting connections and reconfiguring relationships were identified in the discussions.

##### Building cross‐cutting connections

The process of building cross‐cutting connections began with showing the trigger video. For some, it was difficult to watch, but nonetheless effective in making the challenges ‘more personal’ [Y02EI] and inspiring collaboration for improvement.

It [trigger video] was kind of hard to watch because it's something that kind of hits home with me, but I thought it was thorough and it got the point across … [and] de‐isolates the way that individuals can feel, …be[com]ing a rallying point for people … of being more inspired to find solutions … [Y02EI]

In the first co‐design round, participants initially worked in small groups by perspective to develop a prototype to inform the transitions protocol in terms of guiding principles (caregivers), youth friendliness (youth) and protocol elements (providers), respectively. Through facilitated brainstorming, each group collectively shared and built upon members' ideas, with the caveat that ‘no idea should be left behind’ until the ideas ‘gelled’ into an improvement concept. Each perspective developed an initial visual prototype, with participants taking turns ‘holding the pen’ as they drew their prototype solution. Prototyping by perspective ‘empowered each group to talk about the issues that they see’, and then ‘warm up’ to brainstorm in a way that aligned with other perspectives [P04EI]. Prototype images helped to bring ‘clarity and understanding’ to what each group was building. [P09EI]

Participants then offered suggestions to improve initial prototypes developed by the other perspectives. One group member presented the initial prototype to the other groups as they passed through the rooms in turn, enhancing the prototype with their perspectives in a ‘carousel’ approach to co‐design. A provider found it ‘super refreshing’ to see each group's agenda in turn and that ‘… it really all dovetailed nicely together.’ [P04EI] Another found that by ‘…going through each room in advance of kind of combining us’, the process allowed ‘the group dynamic to just continually form around the ideas, as opposed to any group dynamics maybe taking over.’ [P07EI] It also enabled youth voices to be heard,

It was really nice to have the youth take the lead and hear from them, and have them kind of lead that process … it went really well. [P09EI]

Following the carousel co‐design enhancements, the entire group met in plenary and strengthened connections as the prototypes were presented and discussed, recognizing areas of overlap in their suggestions. A caregiver explained that ‘it was inspiring and interesting to see the other groups ideas. There was a similarity with all of them.’ [CG06EI] During this discussion, a caregiver spontaneously shared,

Can I just verbally voice how impressed‐‐ without getting emotional because I do that… I was so inspired. This was the first time I've been with a group that was led by youth or those involved and I thank you. I thank you for that. [CG08CD]

This was followed by a round of applause in the first visible demonstration of the developing feeling of transcendent unity.^34^

Following a networking break, participants worked in mixed groups in the next co‐design round, creating opportunity for frank discussion of differing perspectives,

…there was a bit more conflict, not to say that there was conflict per se. Different generations played a role. The adults are scared, the service providers are unsure, and everyone has something different on their mind, so things can get overwhelming, but overall things went well. And it was necessary, I don't think it shouldn't have happened. [Y03EI]

A youth described gaining ‘new understanding [of] what my mom went through,’ and being ‘brutally honest’ with other caregivers, in a way that they could not be with their own mother. [Y‐AR] A caregiver explained that being detached from the immediate crisis, they could listen ‘to other stories [and]… hear what is going on’, in a way they could not when the primary focus was on ‘What do I need to do for my child?’ [CG‐AR].

##### Reconfiguring the relationship

All prototypes acknowledged the tumultuous changes that youth experience in their lives and service systems, and used metaphors to restructure relationships: placing youth at the centre of a solar system (prototype 1); creating an open tomato cage to support youth to flourish (prototype 2); building a garden path of evolving supports as youth develop (prototype 3); and hand‐holding for continuity across services and supports during transitions, with the youth as the ‘star’ (prototype 4).

I found some of the ideas … really interesting. Like the passports that youth came up with. And even the idea of the growing tomato plant … [I] found it incredibly interesting to consider things from those perspectives. [P07EI]

At the end of the mixed group process, everyone had a chance to comment on the prototypes, clarify different elements and add new ideas during a plenary presentation, so that ‘…everyone was on the same page before leaving,’ [Y01EI]. Youth felt empowered when their ideas were supported and built upon by the whole group.

We actually had the idea of a youth passport and the idea grew and grew and got tweaked and we got a cool amalgamation at the end of it … It was nice to face the group and see their heads nodding in agreement. [Y03EI]

Again, participants felt a sense of transcendent unity,

… you could feel the energy in the room ‐‐ I mean, it was a great feeling because it's like everyone was uniting to actually do something. We do lots of talking in our agencies and amongst ourselves about what needs to happen, but I think it was a great kickoff because it really sort of underlined the energy in the room in that we have a common goal here and we're all actually doing something about it. [P04EI]

**FIGURE 5:**
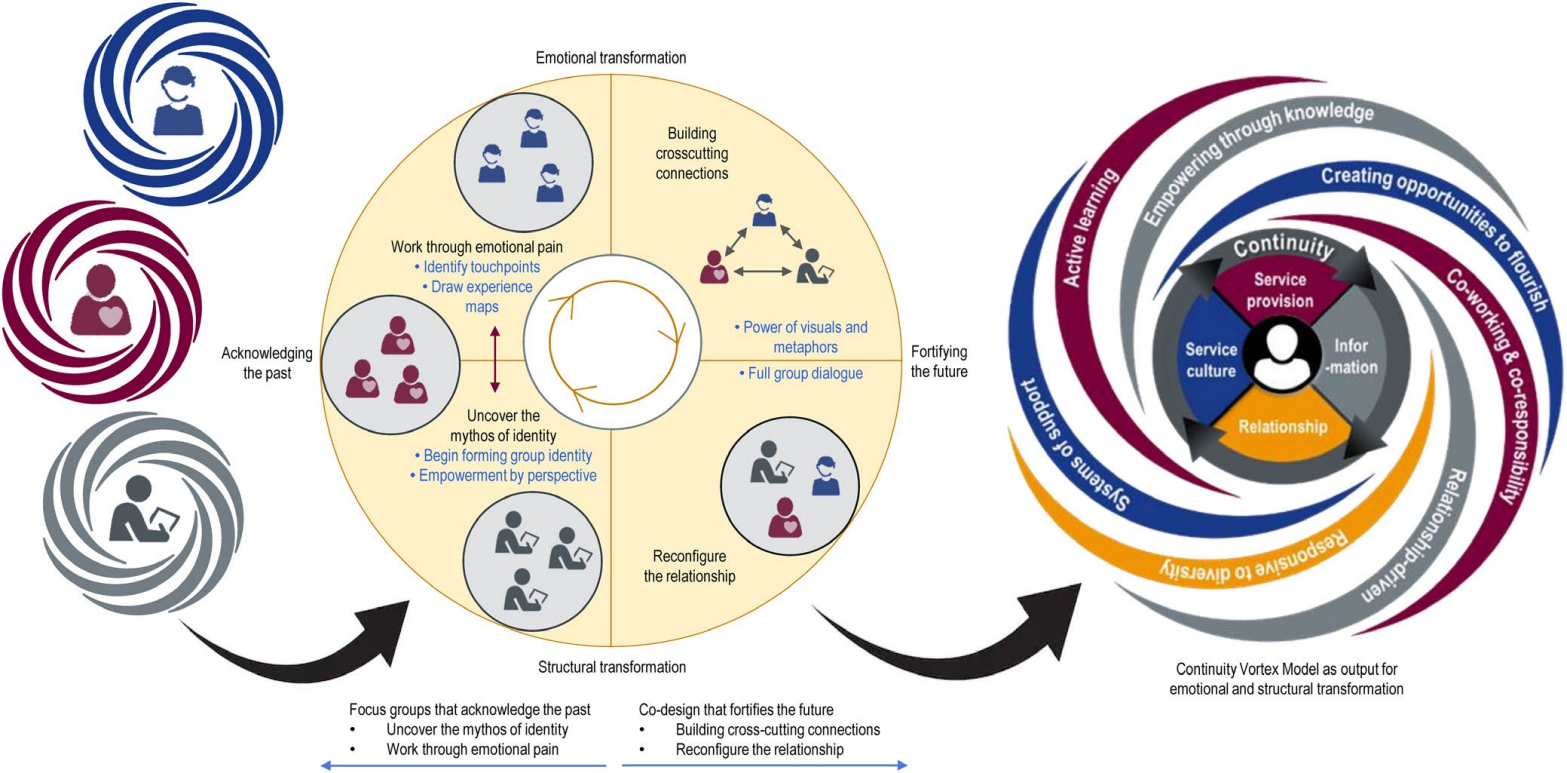
Structural transformation through an EBCD/ID process

Following the co‐design event, the research team developed the continuity vortex (CV) model drawing from the prototypes and deliberations (see Figure 5) as a basis for structural transformation. The CV model reflects the calm stability that youth are seeking within the vortex of change they are experiencing at this developmental stage and during the transition process. It places youth at the centre, and provides continuity of service delivery, relationships, information and service culture, to achieve stability within change. ‘Energy flows’ fuel system‐wide transformation by creating opportunities for youth to flourish, through systems of support that are relationship‐driven, promote active learning, empowerment through knowledge, co‐working and co‐responsibility, and are responsive to diversity. The final model received strong endorsement from a youth,

The Continuity Vortex model that came out of the event truly is a composition of all three parties that attended the event. [Y‐AR]

### 3.2 Achieving emotional and structural transformation

The ultimate aim of the ID process is emotional and structural transformation. From an emotional perspective, it was ‘imperative to address the frustration that each group is experiencing with the current system.’ [CG‐WR] From an emotional perspective, many youth and caregivers described the EBCD process as providing hope through learning they were not alone in their difficult experiences. Some participants gained insight they might not have from their own family members. One caregiver appreciated hearing ‘from other youth experiencing the pain and frustration that my child was feeling, but was unable to express to me’. [CG‐WR] This meant the parent ‘was able to comprehend more, once the personal component was removed.’ [CG‐WR]

From a structural perspective, one youth described the current system as ‘… youth and caregivers and service providers are in different rooms and only connect with tiny windows but can't always see through at the same time’ [Y03EI]. In Figure 5, we illustrate the structural transformation that occurred during our EBCD process, with youth, caregivers and providers initially feeling alone and spinning in a whirl of developmental changes^40^ and through the staged EBCD/ID processes reaching a vision for structural transformation with, ‘The conclusion … that we are all a part of the same team with the same goal in mind, of removing the feeling of helplessness.’ [Y‐WR]

## 4 DISCUSSION

Shapiro^34^ likens the clash of identities in emotionally charged conflict to the earth's shifting plates which typically go unnoticed, until they collide. Such clashes can be destructive, like an earthquake, or by combining ‘identities into a whole greater than the sum of the parts’ (p. 138)^34^ can create a mountain of new strength and stability. Our research suggests that the EBCD process led participants to achieve transcendent unity where there was a history of difficult relations. Applying the ID lens revealed how participants understood their identity in relation to others in the system, worked through their emotional pain, built cross‐cutting connections and reconfigured relationships by ‘synthesizing identities’ during the EBCD process (p. 196).^34^ The CV model moves youth from feeling like victims to being the centre of the relationship; caregivers from exclusion and guilt to inclusion as a supportive resource; and providers from having their hands tied to having an active role in reshaping the transitions process.

We identified three key themes in the EBCD process that facilitated ID. First, the phased approach of EBCD offers an opportunity to work through select ID activities within one's own perspective before working across perspectives. Working through emotional pain and identity mythos mostly occurred in the diagnostic phase (focus groups), where participants felt empowered by discussing touchpoints and learning they were not alone in their experiences. Making cross‐cutting connections and reconfiguring relationships mostly occurred in the intervention (co‐design event) phase. At the same time, consistent with the ID model, there was non‐linearity in moving repeatedly through these processes during other EBCD phases. For example, some participants gained additional insight on painful experiences during discussions with other perspectives during co‐design activities. Building connections began in the separate focus groups as individuals' experiences of being alone and blamed (youth), shut out and guilty (caregivers) and having their hands tied (providers) were recognized as common. This may have helped in building cross‐cutting connections at the co‐design event.^36^ We anticipate various ID processes will be revisited during subsequent EBCD stages where details of CV model implementation are co‐designed by participants.

Second, a gradual approach to integration of groups during EBCD prototyping activities was viewed as an opportunity for fulsome discussion and finding voice in one's own group before working across perspectives. Like the separate focus groups, the initial carousel approach provided a ‘safe space’,^41^ free of judgement to work through pain and develop initial solutions that embodied the core issues of each perspective, before layering on other perspectives. Participants strongly favoured this layering approach and were more open to hearing other perspectives as they built upon each other's work. Plenary sharing demonstrated commonalities that contributed to the experience of transcendent unity witnessed by unanimous applause and later discussed in language consistent with the ID model, such as shifts in ‘energy in the room’, and everyone working ‘toward a common goal’. Achieving unity created a ‘brave space’ that enabled more challenging dialogue in mixed groups that built mutual understanding,^41^ followed by the final plenary discussion that re‐established transcendent unity.

Third, the power of prototyping^31^ in bringing divided groups together was dramatically apparent. Following a brief yet compelling acknowledgement of past pain through the trigger video, participants quickly moved towards fortifying the future through prototyping. As one provider observed, it was easier to focus on visionary metaphors than the details of implementation, which could be divisive.^14^, ^42^ This suggests that a continued focus on ID concepts may benefit the next EBCD stage implementation processes. Furthermore, a policy‐maker and co‐author (JG) found the exercise of exploring the metaphors inherent in the prototypes to be incredibly valuable in understanding the nuance of the messages being communicated by the participants, which is critical to inform policy. For example, caregivers had initially conceptualized a stake with ties that offer stability and consistency while youth developed, but the ties were perceived by youth as too restrictive. Youth preferred an open tomato cage that they could grow out of. This translated into key elements of the CV model: adopting a strength‐based approach that empowers and builds capacity in youth and caregivers; fostering opportunities for youth to flourish; and providing flexibility and appropriateness to each youth's context.

When developing EBCD, Bate and Robert stated that understanding experience ‘… requires an understanding of the interaction and relationship between the user and that service’ (p. 309).^2^ It is therefore not surprising that relational conversion may be needed when bringing together groups where relations have been fractured in the past. In our case, youth who have felt isolated and blamed, caregivers who have felt shut out of care conversations and providers who have felt constrained by siloed and under‐resourced service systems were vocal about the need for relational conversion across perspectives and systems. As participants described, the staged EBCD process resulted in a vision for emotional and structural transformation that creates ‘…positive connection and interaction between the person and the service,’ (p. 309)^2^ which is the end goal of experience‐driven health system improvement according to Bate and Robert.

## 5 IMPLICATIONS FOR RESEARCH

Researchers may benefit from building the ID principles and steps into their own engagement processes when working with mental health service users and caregivers, given the risk of emotionally charged dynamics. For example, while the mythos of identity of each group emerged organically in our processes, this could be made more explicit. Each group could begin by generating their own persona, to visually represent their relationships with others in the system and share this in subsequent ‘rounds’ of discussion with other participants. Similarly, explicit efforts to bring the groups into alignment could include relational prototyping with pre‐post–measurement of perceived alignment. Once ideas for relational change are addressed, further co‐design objectives could be progressed.

Another lesson for mental health researchers was that for many participants, it was easier to build mutual understanding once they had time and distance from immediate crises, and when working with participants of different perspectives who were not immediate family members. This can be an important consideration in designing sampling and recruitment processes.

Finally, while the focus of acknowledging the past in ID is on working through past pain, EBCD captures touchpoints as both emotional highs and lows. Injecting the high points in past experience into deliberations may assist in coming into alignment and envisioning a future of improved relations among historically divided groups, which is consistent with the literature on influence of positive emotions on negotiation.^43^

### 5.1 Strengths and limitations

This work makes an important contribution to the engagement literature by exploring the interpersonal and intergroup dynamics that occur when engaging youth, caregivers and providers in mental health research using the EBCD approach. It also shows how the steps of the ID model align with two key EBCD processes, along with the benefits of a gradual approach to integrating groups in co‐design, and the helpfulness of prototyping. A limitation of this work is that it is based on a retrospective analysis of a single case. A challenge in examining subjective experiences of relational shifts retrospectively was that initial questionnaires and activities were not explicitly designed to probe the ID concepts. Nonetheless, these were witnessed in the data from the EBCD process and were validated in the author and written reflections. To further enhance rigour, the ID themes were analysed based on a guiding conceptual framework using a pattern matching approach that allowed for emergent themes, and were triangulated across multiple data sources and perspectives. Furthermore, this study builds upon a programme of research in which similar shifts towards integrative dynamics were subjectively experienced during EBCD processes. Nonetheless, it is not known to what extent the findings from this case can be generalized to other contexts.

## 6 CONCLUSION

Our findings suggest that the EBCD processes aligned very well with the ID model in this case, suggesting how harmony can be built among groups with a history of emotionally charged conflict. Mental health researchers may benefit from giving explicit consideration to the ID principles and steps when planning and executing engagement activities. In particular, it is essential to create an open, compassionate and cooperative mindset which allows not only a ‘safe’ but a ‘brave space’ for dissonance in deliberations to occur. Engagement activities that strive for harmony, rather than victory, feature gradual integration and prototyping, may not only reduce the risk of ‘us vs them thinking’, but may also promote integration across perspectives and a fundamental restructuring of relations among participants where there has been prior discord.

## CONFLICT OF INTEREST

None declared.

## Data Availability

The data that support the findings of this study are available on request from the corresponding author. The data are not publicly available due to privacy or ethical restrictions.

## APPENDIX 1

### Interview guide for author reflection webinar

1. How would you describe the atmosphere in the room at: (1) the focus group you attended; and (2) the co‐design event?
2. What was your experience of people working through emotional pain at these events?
3. To what extent do you think these processes helped in uncovering and working through how each group (ie youth, family/caregivers, service providers) saw themselves (ie the identity they might be feeling, eg victim, saviour, wise person, other?)
4. To what extent did these processes help to build connections across groups (ie youth, family/caregivers, service providers) and reveal the extent of alignment among them?
5. To what extent did you feel these processes changed the relationships among the groups (ie youth, family/caregivers, service providers)?
6. To what extent do you feel the Continuity Vortex model that came out of the process will change the relationships among the groups (ie youth, family/caregivers, service providers)?

## APPENDIX 2

### Codebook ‐ integrative dynamics

Nodes

**Table:**

Name	Description
Building cross cutting connections	This is a higher level node linked to the ID model. It includes statements of building understanding across perspectives.
Attune to connection	This includes statements that recognize shared understanding or meaning, and other connections across perspectives.
Strengthen relations	This captures any statements that pertain to building stronger relations across perspectives.
Empowering	This captures comments about whether participating in the EBCD process was an empowering experience.
Ensuring safety	This node captures statements regarding anonymity of participants creating a sense of safety in presenting negative feedback.
Logistics	This is a higher level node that captures comments in the evaluation data about logistical considerations.
Enough time	This captures references to wanting more time (or not) in conducting various activities.
Facilitation	This captures comments about the effectiveness of facilitation.
Feeling appreciated	This captures comments about the extent to which the participants felt their contributions were appreciated.
Focus on the positive	This captures any appreciative statements about how the co‐design event focused on improving the system for the future.
Providing background materials	This includes statements about whether having background material to refer to in advance of the events was helpful or not.
Moments of integration	This node includes any ‘aha’ moments where participants describe how the process inspired them, or brought them together in ways they had never experienced before.
Nonlinearity	This captures the back and forth iterative process of working through each of these steps, and emotional responses in particular.
Open mindset	This includes statements about whether a positive, open mindset existed at the co‐design event.
Path to harmony comprises both past and future	This includes statements about the importance of acknowledging the past as well as thinking through new ways of working together in the future.
Reconfiguring the relationship	This is a higher level node that captures comments about how relationships need to/are changing between participants.
Allowing for flexibility as we restructure relationships	This includes statements about the importance of flexibility in the solutions including the need for an individualized approach.
Envision scenarios for co‐existence	This captures examples which include changing extent of family involvement, information‐sharing to inform families; involving youth in every decision; and service providers getting to know each other across organizations.
Power of prototypes and metaphor in reconfiguring	This captures examples of how the use of visuals/metaphor facilitates building appreciation and stronger relationships among participants.
Synthesized identities	This includes statements where identity elements of each group are reflected in the final solution, while allowing for remaining areas of difference (like the centre of a Venn diagram).
Structural transformation	This is a higher level code that captures structural changes being proposed in a number of subdomains.
Accountability	This includes statements about the need for more accountability in the system.
Common approaches across services	This includes statements about the need to have a more common approach between child and adult services.
Improving communication	This includes statements about the need for improved communication across services and stakeholder groups.
Informational restructuring	This captures statement about the need for ways of restructuring information flows among the various groups and services.
Reducing barriers	This includes statements about the need to improve financial, transportation, waitlists as other barriers to smooth transitions.
Structuring the event in stages	This includes statements about whether it was helpful to work together in a step by step process with gradual integration of groups and perspectives.
Uncovering the mythos of identity	This includes data where the personal significance of the issue is uncovered, including, how participants of each perspective view each other. For example, service providers may see each other as the ‘protectors’ of the youth's confidentiality at all costs. This is the higher level code for all 3 perspectives.
Caregiver identity statements	This includes caregiver statements that reveal how they see themselves in relation to others in the system.
Service provider identity statements	This includes statements revealing how service providers see themselves with respect to others in the system.
Youth identity statements	This includes statements that reveal how youth see themselves in relation to others in the system.
Working through emotional pain	This is a higher level code that captures any themes pertaining to emotional pain that were discussed and worked through (intentionally or not, through the activities) consistent with the ID model.
Initial problems	This includes statements about problems in transitions in the current system or in relationships among perspectives.
Not feeling alone	This includes statements about the benefits of hearing from others with similar experiences during the deliberations.
Offering hope	This includes statements about how EBCD processes/activities provided participants with a sense of hope.
Witness and appreciate each other's pain	This includes statements where there is evidence of people interacting to hear and support each other in their pain (past or current) relating to youth mental health transitions.
